# MEDIATORS OF GENETIC AND ENVIRONMENTAL MECHANISMS ON SELF-RATED HEALTH

**DOI:** 10.1093/geroni/igz038.159

**Published:** 2019-11-08

**Authors:** Catalina M Zavala, Carol A Prescott, Susan Lapham

**Affiliations:** 1 University of Southern California, Los Angeles, California, United States; 2 American Institutes for Research, Washington, District of Columbia, United States

## Abstract

Self-rated health (SRH), an individual’s assessment of their own health status, is associated with older adults’ chronic and acute health conditions, as well as mortality. Assessments of SRH indicate individual’s global health is likely multifaceted. Level of education, particularly amount of post-secondary schooling, is associated with better SRH. Other indices of socioeconomic status (SES) such as income and wealth, have varying associations with SRH partly dependent on relative deprivation (e.g. Gini Index). The current study utilized data from 2,500 members of the Project Talent Twin and Sibling (PTTS) Study interviewed as adolescents in 1960 and followed up 54 years later. In 2014, participants were, on average, 70 years of age. Women comprised about 54% of the sample. We examined rearing family wealth, years of education, and functional independence as mediators of variance in SRH. Mean-level results indicated small positive associations between SES and SRH. Activities of Daily Living (ADL) accounted for about a quarter of variance in SRH, with higher functional independence predicting better SRH. Biometric analyses indicated that family wealth had small mediation effects on SRH via familial-environment (S) influences. Education mediated individual-specific (E) environmental influences. Functional independence (measured by ADL) mediated SRH via both additive genetic (A) and E influences. After adjusting for overall effects of sex, age, and specified mediators, a large portion of remaining variation in SRH was due to individual-specific (E) environmental influences. Current results suggest complex underlying genetic and environmental mechanisms contributing to an older adult’s assessment of their own health.

